# Anticoagulation Quality and Complications of using Vitamin K Antagonists in the Cardiac Surgery Outpatient Clinic

**DOI:** 10.5935/1678-9741.20160055

**Published:** 2016

**Authors:** Mário Augusto Cray da Costa, Lucas Kraeski Krum, Juliana da Silva Geraldino, Marcelo Derbli Schafranski, Ricardo Zanetti Gomes, Elise Souza dos Santos Reis

**Affiliations:** 1 Universidade Estadual de Ponta Grossa (UEPG), Ponta Grossa, PR, Brazil and Santa Casa de Misericórdia de Ponta Grossa, Ponta Grossa, PR, Brazil; 2 Universidade Estadual de Ponta Grossa (UEPG), Ponta Grossa, PR, Brazil

**Keywords:** Anticoagulants, Prostheses and Implants, Atrial Fibrillation

## Abstract

**INTRODUCTION:**

In patients with mechanical prosthetic heart valves or atrial fibrillation requiring anticoagulation to prevent thromboembolic events, several factors influence adherence and anticoagulation complications.

**OBJECTIVE:**

To evaluate the factors that interfere with the quality and complications of anticoagulation with vitamin K antagonists.

**METHODS:**

A retrospective cohort study of 100 patients, in the period from 2011 to 2014, was performed. Anticoagulation conditions in the last year, regarding the presence of complications (embolisms/bleeding) and inadequate treatment were assessed: achievement of less than 8 annual prothrombin times and International Normalized Ratio outside therapeutic target in more than 40% of prothrombin times.

**RESULTS:**

There were 31 complications (22 minor bleeding without hospitalization and 9 major complications: 7 bleeding with hospitalization and two emboli); 70 were with International Normalized Ratio outside the target in more than 40% of the tests and 36 with insufficient number of prothrombin times. Socioeconomic factors, anticoagulant type and anticoagulation reason had no relationship with complications or with inadequate treatment. There were more complications in patients with longer duration of anticoagulation (*P*=0.001). Women had more International Normalized Ratio outside the target range (OR 2.61, CI:1.0-6.5; *P*=0.04). Patients with lower number of annual prothrombin times had longer times of anticoagulation (*P*=0.03), less annual consultations (*P*=0.02) and less dose adjustments (*P*=0.003). Patients with longer duration of anticoagulation have more complications (*P*=0.001).

**CONCLUSION:**

There was a high rate of major complications and International Normalized Ratio was outside the goal. Less annual prothrombin times was related to longer duration of anticoagulation, less annual consultations and less dose adjustments. More major complications occurred in patients with longer duration of anticoagulation.

**Table:** 

**Abbreviations, acronyms & symbols**	
AF	= Atrial fibrillation
INR	= International Normalized Ratio
OR	= Odds ratio
PT	= Prothrombin time
SUS	= Unified Health System

## INTRODUCTION

When the surgeons need to replace a heart valve with a prosthesis, they should take into account the life expectancy of the patient and the risk of embolism to decide on the use of biological or mechanical prosthesis^[1,2]^. Still, in a patient with atrial fibrillation (AF) it is necessary to weigh the risks and benefits of anticoagulation^[3,4]^. Important analysis tools such as CHADS and CHA_2_DS_2_VASc scores determine the risk of embolic event setting points according to the comorbidities associated with the AF, on the other hand, the HAS-BLED index establishes the risk of bleeding in these patients^[5-7]^. Such tools help in decision making, but do not take into account socioeconomic and cultural factors that gain expression especially in populations predominantly composed of Brazil's public health system users, called Unified Health System (SUS).

There is not a calculation tool either that safely guides surgeons about the risk of thromboembolic and bleeding complications in patients with mechanical prostheses against the risks of multiple reoperations in patients with bioprosthetic valves. Studies have not demonstrated superiority of one type of prosthesis in relation to each other in terms of mortality, and the sensitivity and experience of each surgeon is responsible for the choice of the prosthesis used when dealing with their patients^[1]^.

Bioprosthetic valves of higher durability and also of very high cost have been developed, but they are not available to the general population, on the other hand, less thrombogenic mechanical prostheses emerged. In the field of pharmacology, new anticoagulants have been used in AF, but with high cost and no good results in patients with mechanical valves, in such a way that in practice vitamin K antagonists should still be used in patients with mechanical prostheses and patients with AF and low purchasing power^[8-10]^.

The most commonly used vitamin K antagonists are warfarin and phenprocoumon, with proven efficacy, although the treatment results need to maintain adequate levels of anticoagulation. In order to control anticoagulation, we use the prothrombin time (PT) test, which is sensitive to reductions of coagulation factors II, VII and X. In 1982, the World Health Organization adopted the international normalized ratio (INR) calibration model, which reports the results of PT in a standardized way^[11]^.

The main complications caused by the use of these drugs are bleeding events, blood dyscrasia, or thromboembolic events resulting from inadequate levels of anticoagulation^[11]^, which occur due to several factors, among them are: patient's difficult access to treatment (related to socioeconomic and cultural factors), and the difficulty of maintaining the INR in the therapeutic values for food and drug interactions^[12-14]^.

Most anticoagulation studies are prospective and it is known that when entering patient in a research protocol the outcomes are different from that observed in clinical practice because the follow-up of these patients is more stringent. Considering that even in prospective studies the rate of complications is not negligible and little more than half of patients achieve therapeutic INR, it is necessary to reflect that in clinical practice the results can be even worse. Thus, the design of this study was performed in order to eliminate the intervention of the researcher on the results and evaluate in a retrospective cohort study what happened to the patient in the last year of anticoagulation until the date of his inclusion in the study.

The aim of this study was to assess the incidence of embolic and hemorrhagic complications in anticoagulated patients with vitamin K antagonists and the relationship with socioeconomic and cultural factors, in addition to assessing the quality of treatment by the number of consultations and the INR number in therapeutic goal.

## METHODS

This is a retrospective cohort study which analyzed 100 consecutive patients using oral anticoagulation with vitamin K antagonists (warfarin and phenprocoumon), who came for consultation in outpatient cardiac surgery and private practice of the cardiac surgery team of Santa Casa de Misericórdia de Ponta Grossa, PR, Brazil, from January 2011 to December 2014.

The study included patients with mechanical prostheses and AF, all under use of warfarin or phenprocoumon who agreed to participate. Patients who underwent surgery in the service, but who made use of anticoagulation control with their respective clinicians, did not participate in the study.

Socioeconomic status, clinical and anticoagulation data in the last year prior to the time of inclusion in the study were collected using interviews with the patient and hospital records consultation. The following negative outcomes considered were: a) embolic or hemorrhagic complications; b) inadequate treatment. It was considered as embolic complication any symptomatic ischemic event; bleeding events were considered as the situations that led to the discontinuation of anticoagulation and may be smaller events (without admission) and major events (requiring hospitalization). Major complications were considered as bleeding requiring hospitalization and thromboembolic events.

It was considered as inappropriate treatment the performance of less than 8 annual PTs (all patients were instructed to perform at least one monthly exam) or when the INR was outside the therapeutic target in more than 40% of the tests. The therapeutic target considered was INR between 2.5 and 3.5 for mitral valve prosthesis^[15]^ and between 2 and 3 for reasons other anticoagulation^[3,15]^.

Regarding the statistical analysis, for continuous variables we used the mean ± standard deviation, compared by the Student's t test, and categorical variables were compared by chi-square test with Yates correction and logistic regression. *P*-value less than 0.05 were considered significant.

All patients signed a written free and informed consent, according to Resolution 196/96. The study was approved by the Research Ethics Committee of the Universidade Estadual de Ponta Grossa, Paraná, Brazil, according to protocol 06532/11.

## RESULTS

The basic characteristics of the sample are presented in Table 1; in which we can observe that there was a similar distribution between the genders, with 73% of patients aged less than or equal to 65 years. As to the place of origin: 66% of the sample resides in municipalities with less than 60,000 inhabitants around Ponta Grossa (regional center city with 311,611 inhabitants, according to the last census) and up to 30% live in rural areas. Regarding education, the group showed a 10% illiteracy rate and 66% only studied up to the 4^th^ grade of elementary school, showing a low education level of the sample. The average number of consultations per year was 3.2±1.6, 52% of patients were SUS users. As anticoagulation indication, 71 were patients with mechanical valve prostheses and 29 had AF; the anticoagulation presented with an average time of 5±3.7 years.

**Table 1:** Basic characteristics of the patients assessed.

**Characteristics**	**Patients n=100**
**SOCIOCULTURAL ASPECTS**	
**Gender**	
Male	54
Female	46
**Age**	
≤ 65 years	73
> 65 years	27
**Location**	
Ponta Grossa (311,611 inhabitants according to IBGE)	34
Other (cities in the region with less than 60,000 inhabitants)	66
**Area**	
Urban	70
Rural	30
**Schooling**	
Illiterate	10
1^st^ to 4^th^ grade	61
4^th^ to 8^th^ grade	14
1^st^ to 3^rd^ year of high school	13
Higher Education	2
**Health insurance**	
SUS	52
Private	48
**Medical consultations/year**	3.2±1.6
**CLINICAL ASPECTS**	
**Indication of anticoagulation**	
Mechanical prosthesis	71
Atrial fibrillation	29
**Type of medicine**	
Warfarin	70
Phenprocoumon	30
**Time of anticoagulation in years**	5.0±3.7
**Number of change dosage/year**	1.6±1.8
**NEGATIVE OUTCOMES: inadequate treatment**	
**Annual PTs insufficient number (< 8)**	
Yes	36
No	64
**Percentage of INR outside the target (inappropriate treatment)**	
More than 40%	70
40% or less	30
**NEGATIVE OUTCOMES: complications**	
**Hemorrhagic complications**	29
Nasal	9 (31%)
Oral	2 (7%)
Genitourinary	4 (14%)
Gastrointestinal	5 (17%)
Bruises	2 (7%)
Skin	7 (24%)
Need for Hospitalization	7 (24%)
**Embolic complications**	2
Ischemic cerebrovascular accident	1 (50%)
Retinal embolism	1 (50%)
**Total of major complications**	9 (9%)

IBGE=Brazilian Institute of Geography and Statistics;

INR=lnternational standard ratio; PT=prothrombin time;

SUS=Unified Health System (Brazilian public health system)

During the study period, 70 patients were outside of therapeutic level (more than 40% of PTs), and 76 performed at least 8 annual PTs. Only 21 patients were considered as proper treatment (therapeutic INR in at least 60% of PTSs and performing at least 8 annual examinations). Nearly a third of patients (n=29) had some kind of bleeding, and of these, seven required hospitalization for treatment of bleeding. There were 2 embolic episodes, one for central nervous system and 1 retinal (Table 1); totaling 9 major complications, namely, those that needed to be hospitalized or had embolism episodes.

**Table 2:** Distribution of patients for the presence of inappropriate treatment.

	**INR outside the target in more than 40%**				**Number of insufficient PTs < 8**			
	**No n=30**	**Yes n=70**	P*	OR (C) P**	**No n=64**	**Yes n=36**	P*	OR (C) P**
**SOCIOCULTURAL ASPECTS**								
**Gender**								
Male	21 (70%)	33 (47%)	0.06	2.61 (6.5-1.0) 0.04	35 (55%)	19 (53%)	0.85	1.08 (0.47-2.45) 0.85
Female	9 (30%)	37 (53%)			29 (45%)	17 (47%)		
**Age**								
≤ 65 years	22 (73%)	51 (73%)	0.96	1.02 (0.39-2.69) 0.96	50 (78%)	23 (64%)	0.19	2.01 ( -5.0 0.82) 0.13
> 65 years	8 (27%)	19 (27%)			14 (22%)	13 (36%)		
**Location**								
Ponta Grossa	10 (33%)	24 (34%)	0.93	0.84 ( -2.07 0.34) 0.71	20 (31%)	14 (39%)	0.58	1.4 (0.59 -3.3) 0.44
Other	20 (67%)	46 (66%)			44 (69%)	22 (61%)		
**Area**								
Urban	18 (60%)	52 (74%)	0.24	0.48 (0.17 -1.33) 0.14	43 (67%)	27 (75%)	0.55	1.46 (0.58 -3.67) 0.41
Rural	12 (40%)	18 (26%)			21 (33%)	9 (25%)		
**Schooling**								
≤ 4 years	22 (73%)	49 (70%)	0.92	1.18 (0.45 -3.07) 0.73	47 (73%)	24 (67%)	0.63	1.38 (0.57-3.36) 0.47
> 4 years	8 (27%)	21 (30%)			17 (27%)	12 (33%)		
**Health insurance**								
SUS	15 (50%)	37 (53%)	0.96	0.89 ( -2.1 0.38) 0.79	32 (50%)	20 (55%)	0.74	0.95 (0.42-2.16) 0.91
Other	15 (50%)	33 (47%)			32 (50%)	16 (45%)		
**Medical consultations/year**	3.1±1.4	3.2 (±1.8)	0.79	NA	3.5 (±1.8)	2.7 (±1.3)	0.02	NA
**CLINICAL ASPECTS**								
**Indication of anticoagulation**								
Atrial fibrillation	8 (27%)	21 (30%)	0.92	1.18 (0.45-3.07) 0.73	16 (25%)	13 (36%)	0.34	1.7 (0.7 -4.1) 0.24
Mechanical prosthesis	22 (73%)	49 (70%)			48 (75%)	23 (64%)		
**Type medicine**								
Warfarin	20 (67%)	50 (71%)	0.81	0.60 (0.24-1.47) 0.27	45 (70%)	25 (69%)	0.93	0.9 (-2.17 0.37) 0.82
Phenprocoumon	10 (33%)	20 (29%)			19 (30%)	11 (31%)		
**Time of anticoagulation**	5.3±3.1	4.9±4.0	0.40	NA	4.4±3.2	6.1±4.3	0.03	NA
**Change of dosage**	1.1±1.3	1.8±2.0	0.08	NA	2.0±2.0	0.9±1.1	0.003	NA
**NEGATIVE OUTCOMES: complications**								
**All the complications**	7 (23%)	23 (33%)	0.47	1.61 (0.60-4.30) 0.34	17 (27%)	13 (36%)	0.44	1.56 (0.65 -3.76) 0.32
**Major complications**	1 (5%)	8 (11%)	0.36	3.74 (0.45-31.33) 0.16	3 (5%)	6 (17%)	0.05	4.06 (1.00-17.30) 0.05

INR= International Normalized Ratio; SUS=Unified Health System; NA=not applicable

* Student t test and Chi-square test with Yates correction;

** Logistic Regression

Table 2 shows the analysis of the factors influencing the suitability for treatment. When comparing the genders, women have more INR outside the target (OR=2.61; C.I.=1.0 to 6.5; *P*=0.04). Sociocultural factors such as age, origin city, urban or rural dweller, education, SUS or private system users, as well as the type of anticoagulant (warfarin or phenprocoumon) and anticoagulation reason (mechanical valve or AF) did not significantly interfere with the occurrence of treatment considered inappropriate (insufficient number of PTs performed and low percentage of therapeutic INR). The average annual consultations was significantly lower among patients who had insufficient number of PTs (*P*=0.02). Regarding anticoagulation time, those with number of PTs smaller than 8 year averaged 6.1 years of anticoagulation *versus* 4.4 years than those who performed satisfactory number of PTs, showing a tendency to worsening of patient care with their INR control over time (*P*=0.03). As expected, those who had number of PTs lower than 8 in one year had less adjustments in dosage (*P*=0.003). The major complications in patients with insufficient number of PTs were 5, since those with sufficient number of tests amounted to 3; generating a tendency to a significant difference between these groups, with an OR of 4.06 (C.I.=0.95-17.40, *P*=0.05). The quality of care was not related to the occurrence of major and minor complications.

Table 3 analyzes the epidemiological characteristics of patients in relation to major complications, and showed that patients with a higher anticoagulation time were more likely to have complications (*P*=0.001). The other factors were not associated with the presence of major complications.

**Table 3:** Analysis of the epidemiological and clinical characteristics of patients in relation to major complications.

**Major complications**				
	**Yes n=9**	**No n=91**	P*	OR (C) P**
**Age**				
< 65 years	6 (67%)	67 (74%)	0.96	1.39 (0.32-6.02) 0.66
≥ 65 years	3 (33%)	24 (26%)		
**Gender**				
Male	5 (56%)	49 (54%)	0.92	0.93 (0.23-3.70) 0.92
Female	4 (44%)	42 (46%)		
**Schooling**				
≤ 4 years	7 (78%)	64 (70%)	0.93	0.68 (0.13-3.47) 0.63
> 4 years	2 (22%)	27 (30%)		
**Area**				
Urban	6 (67%)	64 (70%)	0.82	0.84 ( -3.62 0.20) 0.82
Rural	3 (33%)	27 (30%)		
**Location**				
Ponta Grossa	4 (44%)	30 (33%)	0.74	1.63 (0.41-6.50) 0.49
Other	5 (56%)	61 (67%)		
**Medical consultations/year**	2.4+1.2	3.3±1.7	0.12	NA
**Health insurance**				
SUS	5 (56%)	47 (52%)	0.82	( -3.39 0.22) 0.83 0.85
Other	4 (44%)	44 (48%)		
**Time of anticoagulation**	8.8±6.1	4.6±3.2	0.001	NA
**Change of Dosage**	1.1±1.8	1.6±1.8	0.43	NA

SUS=Unified Health System; NA=not applicable

* Student t test and Chi-square test with Yates correction;

** Regression

## DISCUSSION

In general, the studies on the anticoagulation are controlled, not depicting the reality experienced daily in doctors' offices and SUS clinics. This study was performed in order to identify some of the factors related to the quality of anticoagulation and possible complications, embolic or bleeding, in patients on oral anticoagulation therapy with vitamin K antagonists, experienced in clinical practice. Thus, we opted for a retrospective cohort study, so that the data collected were related to one year before the patients entered the study, so without interference from the researchers control. The study included patients with regular monitoring in the outpatient cardiac surgery, whose anticoagulation is under the supervision of the surgical team. Patients who underwent surgery in the service but who were followed-up by their respective clinicians were excluded from the study. That is the reason for the relatively small number of patients included.

AF, a common arrhythmia, with an estimated prevalence of 0.4% in the general population^[16]^, was present in 29 patients studied. It is estimated that it is responsible for about 5% of the annual incidence of cerebrovascular accident worldwide^[17]^, and current evidence suggests that anticoagulation reduces the incidence of about 70 to 80%^[16,17]^. Although new anticoagulants have shown effectiveness equal to vitamin K antagonists and with the advantage of presenting less bleeding and exempt the PT exams, the cost of these medications still does not allow the use indiscriminately and have not been approved for use in patients with valvular heart disease or prostheses; as the study was performed at a heart surgery clinic, the possibility of use of new drugs is reduced, because of the predominant population of patients with prostheses and valvular heart disease. New studies attempt to assess the use of new anticoagulants in patients with valvular or mechanical prosthesis; but none showed benefits, some show even worse results compared to vitamin K antagonists^[9,10]^.

In patients with valvular prosthesis, anticoagulation therapy varies according to the type and position of such device, being mandatory in patients with mechanical prostheses^[9,16]^. Although warfarin and phenprocoumon reduce thromboembolic complications in patients with AF or mechanical heart valves, the effective management is complex and bleeding events are the main complication of this class of drugs^[18]^, data that are consistent with the findings of this cohort study, in which the number of bleeding episodes was high (29%); if we consider only the major events, the rate is still higher than the literature: 2 embolic events and 7 bleeding requiring hospitalization.

In a developing country like Brazil, access to health care is not always easy. Often, patients do not perform all the necessary examinations, or they do not perform as often as necessary so that they can get good therapeutic results. The average annual consultations found in this study was 3.2±1.6, lower than four annual visits recommended by the team. The Brazilian guidelines of valvular heart disease makes no recommendations as to the time interval between postoperative visits, European guidelines recommend intervals of six months; in this service patients usually are re-evaluated every three months because it is a time considered safe by the group to keep good surveillance in anticoagulated patients^[4,19]^. A national study of anticoagulation in specialized clinics shows an even higher frequency of consultations^[18]^. Data from national and international literature indicate that about 40% to 50% of anticoagulation therapy in patients are outside the target^[17]^. These figures are even worse in this sample, 70% of the examinations were outside the desired level.

One possible explanation for the high number of patients outside the therapeutic level and for major bleeding index may be the fact that this study is a sample of reality, in which the patients were not noticed they were part of a prospective study, as in large clinical trials.

Better anticoagulation control has been related to the educational level of the patients^[20]^, but in this series patients who studied four years or more did not have better quality of treatment, either by the number of tests performed or the percentage of INR in the therapeutic range.

Little is known about the extent to which socioeconomic conditions may influence the risk of bleeding. Some data suggest that low social status is associated with an increased risk of bleeding in the general population, as well as those receiving anticoagulants. The lower socioeconomic status is associated with less access to health^[9]^. Cressman et al.^[9]^ divided the Canadian population into quintiles by income, classified according to the average income of the neighborhood where the patient lived and found that patients in the quintile with the lowest income had higher risk of bleeding than those in the highest income quintile (adjusted risk ratio 1.18, 95% CI 1.12-1.23). In secondary analysis, they identified a similar association between socioeconomic status and risk of fatal bleeding with patients in the lowest income quintile having 28% more likely to die of bleeding associated with warfarin than those in the quintile with the highest income (adjusted risk ratio 1.28, 95% CI 1.11-1.48). In an analysis by bleeding subtype, they found that individuals in the quintile with the lowest income had an increased risk of gastrointestinal bleeding (adjusted hazard ratio 1.18, 95% CI 1.10-1.27) and other bleeding subtypes (adjusted hazard ratio 1.20, 95% CI 1.12-1.29) than those in the highest income quintile, with lower degrees of risk among patients in quintiles 2, 3 and 4. Interestingly, however, they found no association between socioeconomic status and risk of intracranial hemorrhage^[9]^. In this sample the socioeconomic aspects, based on the monetary status were not studied. But when considering patients from the private system and the public health system, there were no differences between complications or quality of anticoagulation. There was, in this sample, great variability in patients' age since older patients with coronary artery disease and AF or degenerative valvular disease were included in the study, as well as younger patients with mechanical valves due to rheumatic fever.

Although the female and old age are considered risk factors for bleeding^[18,20]^, there was no significant difference in patients aged over 65 or between men and women, which was also found in another observational study^[21]^. But it was observed that women had less percentage of therapeutic INR than men.

In this study, we can be observe that in the group that had insufficient number of TP in a year had longer anticoagulant time and fewer consultations in a year, which also resulted in a smaller number of changes in posology; highlighting the difficulty of maintaining an anticoagulation treatment in our country, especially with the passage of time when patients become less careful with their anticoagulation. Avila et al.^[15]^ showed that patients with lower anticoagulation time presented more stable INR than those with long-term use of anticoagulants.

An important point of this study was that the group that had insufficient number of PTs tended to a greater number of major complications (OR:4.06, CI: 1.01-17.40; *P*:0.05); showing that inadequate control of therapy increases the risk of hospitalization and bleeding and thromboembolic events in this group. Regarding anticoagulation time, patients who had major complications were twice as long than those who had no complications (*P*=0.001), noting that the higher anticoagulation time the greater the negligence on the part of the patient to treatment and consequently increased number of complications.

Adherence to treatment with oral anticoagulation is one of the most important factors for the achievement of the optimal level of anticoagulation^[21]^, but the factors that lead to poor adherence have been poorly understood^[14]^. Some listed factors relate to the patient, disease, treatment, health services and social support^[14]^.

In view of the various factors that affect the response to warfarin^[18]^, including the intake of vitamin K (more present in some foods, such as avocados, broccoli, spinach and cauliflower^[16]^), adherence to treatment^[22]^, genetic factors, drug interactions^[23]^, age^[15]^, comorbidities, socioeconomic factors^[24]^, among others, becomes necessary the publication of more studies to investigate these factors in order to reduce the incidence of complications related to anticoagulation, yet very common in our environment.

Treatment of anticoagulation in our country is based on data from other countries. With this study we note the need to expand national studies, as the factors that influence adherence to treatment have their local characteristics.

## CONCLUSION

In our sample the rate of major complications and INR outside the therapeutic target was high. Female patients had less INR in therapeutic goal. Fewer annual PTs were related to longer duration of anticoagulation, less annual consultations and less dose adjustments. More major complications occurred in patients with longer duration of anticoagulation. It is, therefore, the longer period of anticoagulation the factor more related to inadequate treatment and complications.

**Table:** 

**Authors' roles & responsibilities**	
MACC	Conception and design study; realization of operations and/or trials; statistical analysis; analysis and/or data interpretation; manuscript writing or critical review of its content; final manuscript approval
LKK	Analysis and/or data interpretation; statistical analysis; manuscript writing or critical review of its content; final manuscript approval
JSG	Analysis and/or data interpretation; manuscript writing or critical review of its content; final manuscript approval
MDS	Statistical analysis; final manuscript approval
RZG	Analysis and/or data interpretation; final manuscript approval
ESSR	Analysis and/or data interpretation; final manuscript approval
